# Application of Prostate Cancer Models for Preclinical Study: Advantages and Limitations of Cell Lines, Patient-Derived Xenografts, and Three-Dimensional Culture of Patient-Derived Cells

**DOI:** 10.3390/cells8010074

**Published:** 2019-01-20

**Authors:** Takeshi Namekawa, Kazuhiro Ikeda, Kuniko Horie-Inoue, Satoshi Inoue

**Affiliations:** 1 Division of Gene Regulation and Signal Transduction, Research Center for Genomic Medicine, Saitama Medical University, Hidaka, Saitama 350-1241, Japan; takeshi.namekawa@gmail.com (T.N.); ikeda@saitama-med.ac.jp (K.I.); khorie07@saitama-med.ac.jp (K.H.-I.); 2 Department of Urology, Graduate School of Medicine, Chiba University, Chiba, Chiba 260-8677, Japan; 3 Department of Functional Biogerontology, Tokyo Metropolitan Institute of Gerontology, Itabashi-ku, Tokyo 173-0015, Japan

**Keywords:** prostate cancer, cell line, patient-derived xenograft, organoid, spheroid

## Abstract

Various preclinical models have been developed to clarify the pathophysiology of prostate cancer (PCa). Traditional PCa cell lines from clinical metastatic lesions, as exemplified by DU-145, PC-3, and LNCaP cells, are useful tools to define mechanisms underlying tumorigenesis and drug resistance. Cell line-based experiments, however, have limitations for preclinical studies because those cells are basically adapted to 2-dimensional monolayer culture conditions, in which the majority of primary PCa cells cannot survive. Recent tissue engineering enables generation of PCa patient-derived xenografts (PDXs) from both primary and metastatic lesions. Compared with fresh PCa tissue transplantation in athymic mice, co-injection of PCa tissues with extracellular matrix in highly immunodeficient mice has remarkably improved the success rate of PDX generation. PDX models have advantages to appropriately recapitulate the molecular diversity, cellular heterogeneity, and histology of original patient tumors. In contrast to PDX models, patient-derived organoid and spheroid PCa models in 3-dimensional culture are more feasible tools for in vitro studies for retaining the characteristics of patient tumors. In this article, we review PCa preclinical model cell lines and their sublines, PDXs, and patient-derived organoid and spheroid models. These PCa models will be applied to the development of new strategies for cancer precision medicine.

## 1. Introduction

Prostate cancer (PCa) is the most common malignancy and the second most common cause of cancer-related deaths among men in western countries [1]. Androgen deprivation therapy is highly effective for treating patients with advanced PCa, because androgen signaling is essential for PCa growth and antiapoptotic ability [2]. However, despite initial response to androgen deprivation therapy, patients develop castration resistance, leading to the recurrence and/or progression of castration-resistant PCa (CRPC) [3]. Although new androgen receptor (AR)-targeted therapies, such as enzalutamide (ENZ) and abiraterone [4,5], and a chemotherapy agent, cabazitaxel [6], have been approved and used for treating patients with CRPC, these drugs are associated with a limited treatment efficacy. Moreover, although the majority of CRPC tumors show AR-dependent growth by activating AR mutations, amplification, or splice variants, up to 10–20% CRPC tumors lose their AR dependence to evade AR-targeted therapy [7]. One manifestation is the transformation from AR-positive adenocarcinomas to AR-negative small cell neuroendocrine prostate carcinomas (NEPCs). Treatment-related NEPCs account for approximately 25% of almost 34,000 cases of lethal PCa diagnosed per year in the United States [8].

**Figure 1:**
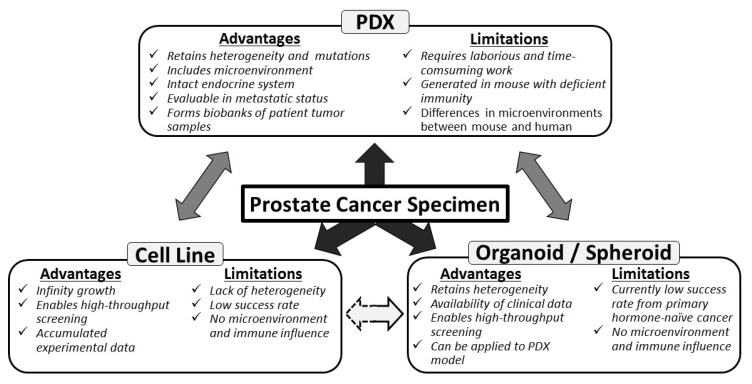
Application of prostate cancer models for preclinical study derived from fresh patient specimens. Each platform has its own advantages and limitations in terms of study design and expected outcome. Traditional cell lines are usually established from metastatic lesions and basically adapted to 2-dimensional monolayer culture. In contrast to cell line platform, recently developed platforms of patient-derived xenograft (PDX) and patient-derived cancer cells (PDCs) in 3-dimensional organoids/spheroids have advantages, as they often retain the characteristics of the original tumor including tumor heterogeneity and complexity. PDX models have advantages, including microenvironment, but limitations due to immunodeficient host background. PDCs can be also applied to PDX models with improved success rates for tumor formation or secondary PDC organoids/spheroids can be regenerated from PDX models vice versa. Organoid/spheroid culture and xenograft models derived from PCa cell lines can also be generated, although these platforms have limitations as they are apart from the actual behavior of clinical prostate cancer without original clinical data.

Various preclinical PCa models have been developed to clarify complex mechanisms underlying treatment resistance of PCa (Figure 1). Three PCa cell lines developed in the 1970s and 1980s, namely LNCaP [9], DU145 [10], and PC-3 [11], are still the most popularly used PCa cell lines in the majority of published studies [12]. Several new original cell lines and their sublines have been developed using various methods, including chemical mutagenesis, genetic alterations induced by castrating host animals bearing xenograft tumors, and viral transformation [13,14]. Because these cell lines show unlimited growth, amenability to high-throughput screening, and formation of xenograft tumors for in vivo testing, they are important resources for identifying the predictors of treatment response and resistance [15,16]. However, the currently available cancer cell lines are associated with some limitations. The established cell lines are selected from specific tumor subsets that grow under in vitro culture conditions. This selection process produces cancer cell lines that do not represent the diversity of human tumors [17]. Moreover, PCa cell lines solely grown in monolayer cultures lack the heterogeneity observed in PCa cells derived from patient tumors [18]. The United States National Cancer Institute has discontinued the use of NCI-60 cell line and its panel of 60 human cancer cell lines, which have been grown in monolayer cultures, from its drug-screening program [19]. Moreover, this institute has recommended the use of patient-derived cancer models for drug screening [19].

Patient-derived xenografts (PDXs) are an important preclinical cancer model for overcoming limitations associated with the use of cancer cell lines and allow investigators to obtain preclinical results that more accurately reflect clinical responses in patients. This is because PDXs grown in immunocompromised mice retain the key molecular aberrations present in patient tumors, including mutations, structural genomic events, epigenetic features, and gene expression programs, which drive their three-dimensional growth [20,21]. The transplantation of human PCa tissues in athymic mice was initially associated with a very poor success rate [22]. Meanwhile, recent technical innovations, including the use of novel highly immunodeficient mice, co-injection of PCa tissues with extracellular matrix (ECM), and transplantation into renal capsules, have improved the success rate of PDX transplantation [23,24,25]. At present, several PCa PDX models have been developed by multiple research groups worldwide [26].

Moreover, transition from monolayer cultures to three-dimensional cultures has increased the successful take rate of patient-derived cancer models [27]. The use of the ECM increases the survival, cellular composition, and differentiation ability of epithelial cells. PCa organoid models have been generated using ECM overlaid with a liquid medium [28]. Gao et al. used this method with PCa specimens to establish seven PCa organoid models [29].

In this review article, we will focus on the in vivo and in vitro PCa models, including original cell lines, original cell line-derived treatment-resistant sublines, PDXs, and patient-derived organoids and spheroids.

## 2. PCa Cell Lines

Since the development of the HeLa cell line, several cancer cell lines have been developed and used for determining mechanisms underlying cancer tumorigenesis and for identifying markers of therapeutic response [30]. Cancer cell lines are associated with some advantages, including infinite proliferation ability and amenability to high-throughput drug screening [31]. The three classic PCa cell lines, namely, DU145, PC-3, and LNCaP, are the most widely used cell lines in PCa research. A PubMed search performed in 2018 by using the names of each of these cell lines (DU145, PC-3, and LNCaP) combined with the term “prostate” yielded 1780, 5475, and 7711 references, respectively. At present, several PCa cell lines have been established from primary PCa tumors, PCa metastases, and PCa xenograft models. In addition, many sublines have been developed from these three classic PCa cell lines, especially the LNCaP cell line. Although the parental LNCaP cells are androgen-sensitive, sublines of these cells, such as LNCaP-abl and LNCaP-LTAD, established by depleting androgen from culture medium, are androgen-insensitive [14,32]. Moreover, several drug-resistant PCa sublines have been developed by incubating the parental cell lines with antiandrogen or chemotherapeutic agents. Here, we have summarized original PCa cell lines derived from primary PCa tumors and metastases, PCa cell lines derived from xenograft tumors (Table 1), and treatment-resistant PCa sublines (Table 2).

### 2.1. Original PCa Cell Lines Derived from Primary Tumors

The 1013L cell line was established in 1978 from a primary prostate tumor [33,34]. Using the 1013L cells with Spongostan, an absorbable hemostatic gelatin sponge, followed by the implantation of this mixture in severe combined immunodeficient (SCID) mice results in the establishment of xenograft tumors [35]. The 1013L cells do not express AR, prostate specific antigen (PSA), and urokinase type PA [36]. Von Bokhoven et al. confirmed that the 1013L cells were established from a unique source [37].

E006AA cell line was established in 2004 from a PCa tissue obtained from a 50-year-old African-American (AA) patient who underwent radical retropubic prostatectomy for treating clinically localized PCa [38]. A culture of epithelial cells from the tumor specimen was established by seeding tumor cells in a low serum-containing medium supplemented with 20 ng/mL cholera toxin to prevent fibroblast contamination. A pure epithelial cell line (designated as E006AA) was established by performing repeated trypsinization, followed by washing to remove detached cells. The E006AA cells show cytokeratin-8, cytokeratin-18, Met proto-oncogene receptor, and AR expression, but low PSA mRNA expression. Initially, these cells were not tumorigenic in nude mice. However, a later study reported that the E006AA cells formed continuously growing tumors in NOG/SCID triple-deficient mice. In 2014, a high tumorigenicity subline called E006AA-hT was derived from a E006AA xenograft tumor isolated from NOG/SCID mice. The E006AA-hT cells show accelerated tumor growth in both nude and SCID mice. One study has reported that AR point mutations may simultaneously produce different loss-of-function and gain-of-function phenotypes in PCa cells [39]. Meanwhile, a non-protein-coding gene locus plasmacytoma variant translocation 1 (PVT1) located on chromosome 8q24 is overexpressed in some PCa. The expression of exon 9 of the PVT1 locus is significantly higher in the aggressive E006AA-hT cells than in other cell lines, suggesting that the PVT1 exon 9 is associated with aggressive PCa in AA men [40].

The RC-77T/E cell line was derived in 2010 from a radical prostatectomy specimen obtained from a 63-year-old AA patient [41]. This patient had clinical stage T3c adenocarcinoma that showed poor differentiation (Gleason score, 7). During passage 4, the RC-77T/E cells were infected with a recombinant retroviral construct LXSN-HPV16E6E7 containing the E6 and E7 genes of HPV-16 and a neomycin resistance gene to induce their proliferation. The RC-77T/E cells produce tumors in SCID mice. These cells express androgen-regulated prostate-specific homeobox gene NKX 3.1, epithelial cell-specific cytokeratin-8, AR, PSA, and p16. Later, a non-malignant prostate cell line RC-77N/E was established from the same patient specimen. Woods-Burnham et al. confirmed that the RC-77T/E cells have single nucleotide polymorphisms feature observed in AA men. [42].

### 2.2. Misclassified Cell Lines

Several studies have reported that some cell lines that were previously believed to be derived from primary tumors are actually derived from cell lines established from metastatic tumors (ALVA-31, PPC-1, BM-1604, and ND-1 cell lines) or other malignant cell lines (JCA-1, PC-93, and PEAZ-1 cell lines) [37,108,109,110].

The ALVA-31 cell line was initially reported to be established from a biopsy specimen of a primary tumor [111]. However, p53 mutation status of the ALVA-31 cells is identical to that of the PC-3 cells. DNA profiling showed that 11 of 13 alleles in the ALVA-31 cells are identical to those in the PC-3 cells [108,109], suggesting that the ALVA-31 cells are derived from the PC-3 cells. The PPC-1 cell line was initially reported to be established from a specimen obtained from a patient with a poorly differentiated prostate adenocarcinoma [112]. Although the PPC-1 cell line was reported to be the first cell line to be derived from a primary prostatic tumor site, later studies reported that this cell line was derived from the PC-3 cells [37,109,113]. The BM-1604 cell line was reported to be established from prostatic adenocarcinoma fragments obtained from a 67-year-old man by performing radical prostatectomy [114]. However, MacLeod et al. performed DNA profiling to show that this cell line was in fact derived from the DU145 cells [110]. In 1992, the ND-1 cell line was reported to be established as a human primary prostatic adenocarcinoma cell line [115]. However, results of DNA profiling and p53 mutation status assessment showed that this cell line was derived from the DU145 cells [37].

The JCA-1 cell line was derived from a poorly to moderately differentiated prostatic adenocarcinoma [116]. However, this cell line has p53 and Ha-ras mutations identical to those in a bladder carcinoma cell line T24, prompting us to investigate possible interrelation between these cell lines. Results of cytogenetics and DNA profiling detected at least 12 structural chromosomal abnormalities that were identical between the T24 and JCA-1 cells [117]. The PC-93 cell line was reported to be derived from a patient with moderately differentiated primary prostate adenocarcinoma [118]. However, a study reported that the PC-93 cell line was identical to the HeLa cell line [37]. The PEAZ-1 cell line was reported to be derived from a prostatic adenocarcinoma specimen obtained from a 63-year-old man with T3a adenocarcinoma by performing radical prostatectomy, and was reported to have several mesenchymal characteristics [119]. However, this line was retracted by the original investigators because it was found to be derived from a fibro-sarcoma cell line HT-1080 [37].

### 2.3. Original PCa Cell Lines Derived from Metastasis Tumors

The DU145 cell line was the first PCa cell line and was derived in 1975 from a brain metastatic prostate tumor of a 69-year-old white man [10,43]. The DU145 cells show low PAP expression and do not show AR or PSA mRNA and protein expression. Moreover, these cells express CK-7, CK-8, CK-18, and CK-19 but do not express CK-5, CK-14, CK-19, and CK-20. Meanwhile, a recent study has suggested that these cells also express CK-5 [44]. Subcutaneous injection of the DU145 cells into nude mice produces tumors that maintain their phenotype and genotype [10]. Moreover, the injection of the DU145 cells into non-obese diabetic (NOD)/SCID mice produces xenograft tumors that metastasize to different organs, including the spleen, lungs, and liver [10,45]. On the other hand, DU145 xenografts in *pfp*^−/−^/*rag2*^−/−^ mice have very weak metastatic potential [46,47]. Metastasis behavior was, therefore, dependent on the host immune status and microenvironments.

The PC-3 cell line was isolated in 1979 from a lumbar vertebral metastatic prostate tumor of a 62-year-old white man [11]. The PC-3 cells are hormone insensitive and do not show AR and PSA expression of mRNA as well as protein but show high TGF-alpha and EGFR mRNA expression [48]. Subcutaneous injection of the PC-3 cells into nude mice produces tumors containing undifferentiated malignant cells. The PC-3 cells also form tumors in female nude mice, suggesting that their growth is androgen independent. A PC-3M cell line was established from a PC-3 xenograft tumor in athymic mouse and is more aggressive than the parental PC-3 cell line [49]. Moreover, four distinct PC-3 sublines have been isolated that preferentially metastasize to the lumbar vertebrae (PC-3 ML cell line), mandibular region of the right cheek (PC-3 MC cell line), rib cartilage (PC-3 MR cell line), and right front knee bone (PC-3 MK line) in SCID mice at approximately 80% efficiency [50]. Recent studies have shown that the PC-3 cells show the characteristics of neuroendocrine or small cell carcinoma compared with those of adenocarcinoma [37,51,52]. Moreover, the PC-3 cells show high expression of two NEPC markers, namely, chromogranin A (CgA) and neuron specific enolase (NSE) [37,52]. Results of immunohistochemical studies have shown that PC-3 xenograft tumor cells show extensive positivity for the NEPC marker NSE compared with LNCaP xenograft tumor cells, which show negativity for the NEPC marker NSE [51]. NEPC tumor cells commonly express CD44, a putative cancer stem cell-associated marker [53]. Results of immunohistochemical studies have shown that the PC-3 xenograft tumor cells show a strong and diffuse positivity for CD44 similar to NEPC tumor cells compared with the LNCaP xenograft tumor cells, which show negativity for CD44 similar to bulk tumor cells derived from prostatic adenocarcinoma [51].

The LNCaP cell line was established in 1980 from a needle aspiration biopsy of a lymph node metastatic lesion obtained from a 50-year-old white man [9]. The LNCaP cells grow well in media supplemented with 2.5–10% fetal calf serum (FCS). These cells are androgen-responsive because they show AR and PSA expression of mRNA as well as protein [54]. The LNCaP cells contain a T877A point mutation in the ligand-binding domain of AR that allows these cells to show an atypical response to steroid compounds [55]. Moreover, the LNCaP cells express CK-8, CK-18, and CK-20 and contain a wild-type (WT) TP53 gene [37,56,57]. Xenografting of a minimum of 3 × 10^6^ cells with Matrigel™ is associated with a modest success rate of 50% and tumor doubling time of 86 hours. Tumor formation occurs at a faster rate and is more efficient in male hosts than in female hosts; however, the growth rate of xenograft tumors is independent of the host gender [12]. PCa sublines described below have been derived from the parental LNCaP cells through different methods, including long-term androgen deprivation, anti-androgen and chemotherapeutic agent treatment, stable transfection, and selection of metastatic cell lines by serial passing in mice.

ARCaP cell line was derived in 1996 from the ascites fluid of an 83-year-old white man with advanced metastatic PCa [58]. The ARCaP cells show low AR mRNA and PSA mRNA and protein expression. Immunohistochemical analysis indicates that the ARCaP cells strongly express EGFR, c-erb B2/neu, and c-erb B3. Moreover, these cells express bombesin, serotonin, NSE, and c-met protooncogene, but do not CgA. These cells also secrete high levels of gelatinase A, gelatinase B, and stromelysin, which are markers of invasive adenocarcinoma with selective NEPC phenotypes. Interestingly, dihydrotestosterone repressed the growth of ARCaP cells in vitro in a concentration-dependent manner. The ARCaP cells show 100% tumor formation efficiency when injected into intact or castrated nude mice. Moreover, tumors derived from these cells show 3-times faster growth in castrated mice than in intact mice.

The MDA PCA 2a and 2b cell lines were derived in 1997 from a bone metastasis tumor of a 63-year-old AA man who had PCa showing androgen-independent growth [59]. Although both these cell lines were derived from two samples of the same specimen, they show different genetic features and phenotypes. Therefore, it is likely that these cell lines are distinct clones isolated by using different culture procedures, or reflect the genetic heterogeneity of the original tumor. These cell lines contain two mutations, namely, L701H and T877A, in the ligand-binding domain of the AR gene. Compared with the LNCaP cells, the MDA PCA 2a cells show significantly low response to dihydrotestosterone (DHT) and R1881 and to other androgens, such as testosterone [59]. One factor that differentiates the MDA PCA 2a and 2b cells is that only the MDA PCA 2a cells express the Bax gene. The integration rate of these cells in mice increases with the use of Matrigel™. In vivo, the MDA PCA 2b cells show a faster growth than the MDA PCA 2a cells; however, only the MDA PCA 2a cells form palpable intraprostatic tumors after 11 weeks [60].

### 2.4. Original PCa Cell Lines Derived from Xenograft Tumors

LuCap 23 cell line series was derived in 1996 from xenograft tumors obtained from a 63-year-old white man with stage DI prostate adenocarcinoma (Gleason score, 8) at autopsy [61]. The patient was treated by administering external-beam radiation therapy to the pelvis, bicalutamide, bilateral orchiectomy, and chemotherapy with adriamycin and carboplatinum. Tumors isolated from three separate metastases in this patient were used to develop three xenograft sublines, namely, LuCaP23.1 and 23.8 (from lymph node metastases), and LuCaP 23.12 (from a liver metastasis). These cell lines show high PSA expression. However, androgen deprivation therapy decreases PSA secretion and tumor size of xenograft tumors derived from these cells. Eventually, tumors derived from these cells become androgen-independent and resume growth in castrated mice. Moreover, subcutaneous xenografts of LuCap23.1 develop spontaneous lung metastases with moderate frequency in *pfp*^−/−^/*rag2*^−/−^ mice [46]. These results suggest that the LuCap 23 cell line series and xenograft tumors derived from these cells show many phenotypic characteristics, including androgen sensitivity, of clinical prostatic carcinoma [62].

The LAPC-4 cell line was established in 1997 from a xenograft model of a femoral PCa metastasis formed in a patient undergoing androgen ablation therapy [63]. These cells show WT AR and PSA expression, and produce tumors after subcutaneous injection of as few as 10 cells into intact mice. However, only a fraction of such injections produce tumors in castrated mice. At present, the LAPC-4 cells are widely used as AR-positive PCa cells [64,65].

The 22Rv1 cell line was derived in 1999 from a xenograft CWR22R isolated from a patient with bone metastasis. The CWR22R xenograft was serially propagated in mice after castration-induced regression and relapse of a parental, androgen-dependent CWR22 xenograft [66]. In nude mice, the 22Rv1 cells form tumors having morphology similar to that of its parental xenograft. Moreover, like the parental CWR22 and CWR22R xenografts, these cells show PSA expression. These cells are of particular interest to investigators researching AR splice variants. AR splice variants that are activated in a ligand-independent manner are assumed to be the main players in hormone-refractory tumor progression [67,68]. Functionally, these AR isoforms are constitutively active and promote the expression of endogenous AR-dependent genes and proliferation of the 22Rv1 cells in a ligand-independent manner [2].

The VCaP cell line was derived in 2001 from a vertebral metastatic lesion through autopsies [69,70]. This metastatic tissue was aseptically xenografted into SCID mice and was later harvested and plated in tissue culture dishes. The VCaP cells show androgen sensitivity and WT AR and PSA mRNA and protein expression. In vivo, the doubling time of tumors derived from the VCaP cells is 10 days in intact male mice, 13 days in castrated male mice, and 23 days in female mice. Because the VCaP cells proliferate in castrated mice, they are suggested to show androgen-independent growth.

The KUCaP cell line was derived in 2005 from an autopsy specimen of liver metastasis obtained from a 64-year-old Japanese male patient who died because of CRPC [71]. The KUCaP cells express AR with a W741L point mutation in its ligand-binding domain. This mutation was also present in the cancerous tissue used for generating the KUCaP cells. The KUCaP cells grow in male nude mice in an androgen-dependent manner; however, treatment of these cells with the antiandrogen drug bicalutamide aberrantly promotes their growth and PSA production [72,73]. Serum- and glucocorticoid-regulated kinase 1 (SGK1), which upregulates bicalutamide-induced AR expression (W741L), is highly expressed in xenograft tumors derived from the KUCaP cells [74].

PC346 cell line was derived from a xenograft model PC346 derived from the transurethral resection of a primary prostate tumor [75]. The PC346 cells are androgen-responsive and show slow growth in a steroid-stripped medium. However, these cells show a 2- to 3-fold increase in growth upon stimulation with a synthetic androgen R1881 but do not show growth upon stimulation with an antiandrogen hydroxyflutamide [75].

### 2.5. PCa Sublines Showing Treatment Resistance

#### 2.5.1. Castration-Resistant PCa Sublines

The LNCaP-abl cell line was established after 41 passages of the parental LNCaP cells in an androgen-depleted medium [32]. The morphology of the LNCaP-abl cells is different from that of the parental LNCaP cells, in that the LNCaP-abl cells form clusters rather than typical uniform monolayers. Moreover, the LNCaP-abl cells show approximately 4-fold higher AR protein expression than the parental LNCaP cells. Furthermore, the basal AR transcriptional activity is 30-fold higher in the LNCaP-abl cells than in the LNCaP cells. Interestingly, treatment with bicalutamide, which inhibits the growth of the parental LNCaP cells, stimulates the proliferation of the LNCaP-abl cells. Bicalutamide treatment also exerts agonistic effects on AR transactivation activity in the LNCaP-abl cells but does not block the effects of androgens in these cells.

The LNCaP-SF cell line is an androgen-independent LNCaP subline that was established by culturing the LNCaP cells in RPMI-1640 medium supplemented with a steroid-stripped serum for a long period (more than six months) [76]. The LNCaP-SF cells show higher UGT2B15 expression and 2.5-times higher glucuronidation activity than the parental LNCaP cells. Moreover, the LNCaP-SF cells show more rapid proliferation in castrated mice than in normal mice. Androgen treatment induces PSA expression but does not affect AR, p21, p27, and cyclin D1 expression in the LNCaP-SF cells [77].

The LNCaP-LTAD cell line was established by culturing the LNCaP cells in a phenol-red free RPMI-1640 medium supplemented with 10% charcoal-stripped FBS for more than 9 months [14,78]. The LNCaP-LTAD cells show high expression of the androgen-regulated gene TACC2, which contributes to the hormone-refractory proliferation of these cells. Moreover, the expression of androgen-regulated lncRNA *SOCS2-AS1* is higher in the LNCaP-LTAD cells than in the parental LNCaP cells. This lncRNA promotes castration-resistant and androgen-dependent growth of the LNCaP-LTAD cells and upregulates androgen signaling in these cells by modulating the epigenetic control of AR target genes [79]. In this paper, the VCaP-LTAD cell line was also established from VCaP cells by a similar method.

The C4-2 cell line was isolated in 1994 from a mouse vertebral metastasis of LNCaP xenografts [80]. To generate the xenograft mouse model, the LNCaP cells were subcutaneously co-injected with MS cells, a bone stromal cell line. Xenograft tumors derived from the C4-2 cells show PSA secretion. In castrated mice, these tumors show progression from an androgen-dependent phenotype to an androgen-independent phenotype upon cellular interaction with bone fibroblasts. In detail, LNCaP subline C4 was derived from castrated mice and produced tumors in castrated mice when co-injected with bone fibroblasts. A second-generation LNCaP subline C4-2 was derived from a chimeric tumor produced by co-inoculating the C4 cells with MS cells in castrated mice. The C4-2 subline was tumorigenic when inoculated into castrated mice in the absence of inductive fibroblasts. Compared with the parental LNCaP cells, the C4-2 cells show low steady-state AR mRNA and protein expression and lose its androgen responsiveness in vitro [80]. Upon subcutaneous or orthotopic inoculation, the C4-2 cells metastasize to the lymph nodes and bones. Another subline C4-2B has been derived from the bone metastasis of the C4-2 cells [81].

#### 2.5.2. Antiandrogen-Resistant PCa Sublines

The PC346Flu1 and PC346Flu2 cell lines were derived from PC346C cells by culturing in an androgen-depleted medium supplemented with 2% charcoal-stripped FCS and 1 µM hydroxyflutamide [82]. These flutamide-resistant cell lines show different AR expression statuses. While the PC346Flu1 cells overexpress AR, the PC346Flu2 cells show a T877A mutation in the AR gene.

The LNCaP-BicR cell line (Takayama et al.) was established by culturing the LNCaP cells in RPMI 1640 medium supplemented with 10% FBS and 10 μM bicalutamide for more than 3 months [13]. Bicalutamide treatment does not inhibit the proliferation of the LNCaP-BicR cells even though it inhibits the proliferation of the parental LNCaP cells. Moreover, the LNCaP-BicR cells show proliferation in the absence of bicalutamide compared with the parental LNCaP cells. Interestingly, the AR-binding sites in the LNCaP-BicR cells, which have been determined by performing bicalutamide treatment, overlap the binding sites of an AR agonist DHT, suggesting that bicalutamide mediates AR recruitment to genomic regions in the LNCaP-BicR cells [13].

The LNCaP-BicR cell line (Liu et al.) was established by culturing the LNCaP cells with increasing concentrations of bicalutamide (1–40 µM) for over 12 months [83]. The LNCaP-BicR cells show significantly increased mRNA and protein expression of AR splice variants, particularly AR-V7. Exogenous AR-V7 expression in bicalutamide-sensitive LNCaP cells confers bicalutamide resistance to these cells. In contrast, AR-V7 knockdown in the LNCaP-BicR cells reverses bicalutamide resistance in these cells.

The MR49F is an ENZ-resistant cell line derived by culturing cells obtained from ENZ-resistant LNCaP xenografts in RPMI-1640 medium supplemented with 5% FBS and 10 μM ENZ [84]. The MR49F cells have been used as an ENZ-resistant PCa model to evaluate new AR-targeting drugs [84,85].

The ENZ^R^ cell line series, which also shows ENZ resistance, was derived from cells obtained from ENZ-resistant LNCaP xenografts [86]. An ENZ-resistant xenograft model (ENZ^R^) was established by injecting the LNCaP cells in intact male athymic mice to produce subcutaneous tumors, followed by the castration of these mice. After tumor recurrence (CRPC), the mice were treated with vehicle or 10 mg/kg ENZ daily. Although the ENZ treatment decreased tumor growth compared with the vehicle treatment, it did not prevent tumor recurrence. The primary PSA-positive ENZ^R^ xenografts also produced nine serially transplanted tumors out of 35 (25.7%) tumors that showed no increase in PSA expression. Different cell lines have been derived from multiple transplanted ENZ^R^ tumors (42D^ENZR^, 42F^ENZR^, 49C^ENZR^, and 49F^ENZR^). The 42D^ENZR^ and 42F^ENZR^ cell lines derived from the PSA-expressing tumors show reduced expression of classic AR target genes and increased expression of canonical transcription factors and markers associated with NEPC. These ENZ-resistant cell lines may be used for examining mechanisms underlying the development of both classic AR-driven and AR-undriven PCa phenotypes, especially the transdifferentiation of ENZ-resistant CRPC to NEPC.

#### 2.5.3. Chemotherapy-Resistant PCa Sublines

(a) Paclitaxel-Resistant PCa Cell Lines

The DU145-TxR and PC-3-TxR are paclitaxel-resistant PCa cell lines established in 2007 by incubating the parental DU145 and PC-3 cells, respectively, with gradually increasing concentrations of paclitaxel for 2 days, followed by culturing in the absence of paclitaxel until the cells showed appropriate growth [87]. The IC_50_ of paclitaxel against the DU145-TxR and PC-3-TxR cells is 34.0- and 43.4-fold higher, respectively, than that against both the parental cell lines. Both the cell lines show cross-resistance to estramustine phosphate, docetaxel, vinblastine, and doxorubicin, but not to etoposide and cisplatin. Knockdown of P-glycoprotein, which is upregulated in resistant cells, by using MDR1 siRNA restores paclitaxel sensitivity of the DU145-TxR cells. Microarray analysis has shown that the C-terminal tensin-like protein (CTEN, tensin 4) gene is downregulated by 10-fold in the PC-3-TxR cells. CTEN knockdown in the PC-3 cells induces paclitaxel resistance, and CTEN overexpression in the PC-3-TxR and DU145-TxR cells restores paclitaxel sensitivity [88]. These cell lines have been widely used to evaluate new therapeutic targets for treating taxane-resistant PCa [89,90,91,92,93,94,95,96].

The PC3PR cell line is a paclitaxel-resistant cell line established from the PC-3 cells [97]. The IC_50_ of paclitaxel against the PC3PR cells is 2577.3 nM and that against the parental PC-3 cells is 8.6 nM. MSK1 knockdown by using miR-148a decreases the paclitaxel resistance of the PC3PR cells, indicating that miR-148a attenuates paclitaxel resistance in the hormone-refractory, drug-resistant PC3PR cells partly by regulating MSK1 expression. Moreover, reduced miR-34a expression confers paclitaxel resistance to the PC3PR cells by upregulating SIRT1 and BCL2 expression [98]. The PC3PR cells have been used to identify new therapeutic targets, including ETS1 and CCR1 [99,100].

The PC-3-Pa cell line was established in 2018 from the PC-3 cells and shows >100-fold higher resistance to paclitaxel than the paclitaxel-sensitive PC-3 cells [101]. Treatment with selenonucleoside (4ʹ-selenofuranosyl-2,6-dichloropurine, LJ-2618) effectively inhibits the proliferation of both PC-3 and PC-3-Pa cells in vitro, with similar IC_50_ values. LJ-2618 treatment suppresses the activated PI3K/AKT signaling pathway in the PC-3-Pa cells. Treatment of a PC-3-Pa cell-implanted xenograft mouse model with 3 or 10mg/kg LJ-2618 effectively inhibits tumor growth by enhancing SKP2 degradation and by inducing p27 expression in the tumor tissues.

(b) Docetaxel-Resistant PCa Cell Lines

The PC-3dR cell line was derived by repeatedly exposing the PC-3 cells to docetaxel in vitro [102]. For producing the PC-3dR cells. the PC-3 cells were serially treated with 0.1, 1, 5, or 10 nM docetaxel for 1 week, followed by no treatment for 1 week. Secretory clusterin expression is 2.5-fold higher in the PC-3dR cells than in the parental PC-3 cells. Combined treatment with an antisense oligonucleotide against the human secretory clusterin and paclitaxel treatment synergistically inhibit the growth of PC-3dR xenografts in nude mice.

The DU145 R, 22RV1 R, and PC-3 D12 cell lines were developed by culturing their respective parental cells with gradually increasing concentrations of docetaxel over 6 months [103]. The cells were continuously maintained in docetaxel concentrations corresponding to its IC_50_ values against the respective parental cell lines. The IC_50_ of docetaxel against the PC-3 D12 cells is 10-fold higher than that against its parental cells. Treatment of the PC-3 D12 cells with BAY 11-7082 inhibitor represses docetaxel-induced increase in NF-κB activity and increases sensitivity to docetaxel. In addition, an analysis of these cells showed that ZEB1 promotes both epithelial-mesenchymal transition (EMT) and docetaxel resistance in these cells by repressing the transcription of E-cadherin [104].

The DU145R and PC-3R cell lines were developed in 2012 and show 2- to 5-times higher resistance to docetaxel than their respective parental cells [105]. The IC_50_ of docetaxel against the DU145R and PC-3R cells ranges from 10 to 15 and from 20 to 22 nM, respectively, whereas that against the DU145 and PC-3 cells ranges from 4 to 5 and from 3 to 5 nM, respectively. Results of microarray analysis have shown that 243 overlapping genes are differentially expressed in both the DU145R and PC-3R cells.

(c) Cabazitaxel-resistant PCa cell lines

The DU145CR and PC3CR cell lines were established by culturing the DU145 and PC-3 cells, respectively, with increasing concentrations of cabazitaxel (0.3–3 µM) for 2 years [106]. The DU145CR cells show resistance to cabazitaxel-induced G2/M cell cycle arrest through ERK signaling activation. An MEK inhibitor significantly inhibits the proliferation of the DU145CR cells. The PC3CR cells show enhanced PI3K/AKT signaling, and a PI3K/mTOR inhibitor exerts a significant antitumor effect on the PC3CR cells.

The DU145-TxR/CxR and PC-3-TxR/CxR cell lines were established by culturing the DU145-TxR and PC-3-TxR cells, respectively, which are paclitaxel resistant (see above), with gradually increasing concentrations of cabazitaxel for 6 months [107]. The DU145-TxR/CxR and PC-3-TxR/CxR cells show 4.4- and 11.8-fold higher resistance, respectively, to cabazitaxel than their corresponding parental cells. The DU145-TxR/CxR and PC-3-TxR/CxR cells also show resistance to cabazitaxel after inoculation in SCID mice. Comparison of cDNA microarray data of the DU145-TxR with DU145-TxR/CxR cells or the PC-3-TxR with PC-3-TxR/CxR cells indicates that many genes are upregulated or downregulated in these cells. The MDR1 gene is upregulated in the PC-3-TxR cells compared with that in the PC-3 cells and is further upregulated in the PC-3-TxR/CxR cells compared with that in the PC-3-TxR cells. MDR1 knockdown restores the sensitivity of both the PC-3-TxR/CxR and DU145-TxR/CxR cells to cabazitaxel.

(d) Cisplatin-Resistant PCa Cell Lines

The LNCaP/C sublines were established by culturing the LNCaP cells with increasing concentrations of cisplatin [120]. This resulted in the development of three sublines, namely, LNCaP/C1, LNCaP/C2, and LNCaP/C3, that show 6.3-, 9.1-, and 22.3-fold higher resistance to cisplatin, respectively, than the parental LNCaP cell line. Moreover, this resistance to cisplatin is associated with the reduced induction of apoptosis [120]. The LNCaP/C3 cells show cross-resistance to adriamycin, 5-fluorouracil, and etoposide, and either no cross-resistance or only weak cross-resistance to taxol and taxotere [120].

## 3. Patient-Derived Xenografts

Exhaustive efforts have been made for years in terms of in vivo tumor formation experiments based on human tumor cell lines, and the usefulness of this model has been shown as exemplified by the evaluation of drug efficacy and the identification of various therapeutic targets for the disease. Nevertheless, the cell line model has a limitation in terms of the in vitro adaptation of cells to culture conditions, which sometimes leads to the discrepancy between the experimental and clinical outcomes. PDXs may more appropriately recapitulate the molecular diversity, cellular heterogeneity, and histology in patient tumors [24,25]. The in vivo use of PCa PDXs helps in evaluating both anticancer efficacy and toxicity, thus providing a therapeutic index of new approaches that need to be evaluated simultaneously [26]. Until the 1990s, the transplantation of human PCa tissue in athymic nude mice was associated with a very poor integration rate of <5% [121]. In 1990, Fridman et al. reported a high integration rate by transplanting small cell lung cancer cells along with Matrigel^TM^ into nude mice [122]. Recent advances in xenografting primary tumors in renal capsules have improved graft survival but not tumor growth [123]. Toivanen et al. reported improved methods to xenograft localized primary PCa tissues by using chimeric grafts with neonatal mouse mesenchyme [124]. Establishment of highly immunodeficient mice has also improved the integration rate and xenograft tumor growth (details explained below) [23]. Although the establishment of PCa PDXs is still challenging, with an integration rate of 10–40% and prolonged latency time, some groups have developed methods to successfully develop serially transplantable human PCa PDXs by using immunodeficient mice [20,21]. In this section, we have summarized PCa PDX models, especially xenograft models, produced by directly xenografting patient-derived tissues into mice (Table 3).

### 3.1. Advancements in the Development of Immunodeficient Mice

Flanagan et al. reported the first immunodeficient nude mouse model in 1966 [162]. These mice lack body hair and are athymic. Moreover, these mice cannot generate mature T lymphocytes, indicating that they cannot elicit an adaptive immune response. Since then, nude mice have been used as recipients for transplanting human cancer cells. In 1983, SCID mice, which show impaired differentiation of both T and B lymphocytes, were generated by Bosma et al. [163]. SCID mice are better recipients of xenografts than nude mice and show successful xenografting of human hematopoietic stem cells and mature blood cells. NOD mice were initially developed as a model of non-obese diabetes mellitus. These mice show complex immunodeficiency features, such as dysfunction of dendritic cells, macrophages, and natural killer (NK) cells [164]. The signal regulatory protein-alpha (SIRPA) polymorphism is also involved in a mechanism of immune tolerance in immunodeficient strains. The NOD-specific SIRPA binds to human CD47, and this binding prevents host macrophages from engulfing human grafts, thereby inhibiting rejection [165]. NOD/SCID mice were established by crossing the NOD and SCID mice. These mice show a high efficiency of accepting malignant human hematopoietic cells and solid tumors [166]. In 2000s, more appropriate recipient mice were established for xenotransplantation. Namely, NOD/SCID/IL2Rγnull mice, also called NOG mice [167] or NSG mice [168], were generated by performing eight backcross matings of C57BL/6J-γnull and NOD/Shi-SCID mice. These mice completely lack NK cell activity and show improved efficiency of accepting PDXs of solid tumors, including PCa. *Pfp*^−/−^/*rag2*^−/−^ mice are completely devoid of functional T- and B-lymphocytes, as well as NK cells. As NK cells are potent defenders against circulating tumor cells, *pfp*^−/−^/*rag2*^−/−^ mouse strain is of particular interest in metastasis research [46,169]. Although establishment of severe immunodeficient mice improved PDX take rate, these models have a limitation of application since metastatic behavior of cancer cells in severe immunodeficient models differ from the clinical situation. Recently, the human immune system can be developed in NSG mice by human haematopoietic stem cells transplantation. This model is called “humanized mouse” and has functional human T cells, NK cells, and monocytes [170].

### 3.2. Patient-Derived Xenograft Models

The Rotterdam PCa models were derived from primary PCa specimens (prostatectomy specimens), transurethral resection specimens, and metastatic lesions (pelvic lymph nodes and scrotal skin) at Erasmus University [22,75,121]. In 1977, the first androgen-dependent in vivo PCa model, designated as PC-82, was established, followed by the development of two androgen-independent in vivo models PC-133 and PC-135 [125]. In 1984, PC-EW, another androgen-dependent PDX model, was established [126]. In 1996, seven human prostate tumor models were established [121]. Histological examination of xenograft tumors derived from these models indicates that these xenograft tumors retain the characteristics of the original patient tumors. Tumors derived from PC-295, PC-310, PC-329, and PC-346 models depend on androgens for their growth, whereas those derived from PC-324, PC-339, and PC-374 models do not require androgens for their growth; however, tumors derived from PC-374 model seem to be androgen-sensitive [121,127].

The LuCaP PDX models were established from primary PCa tumors or PCa metastases derived from operative specimens and metastatic CRPC samples obtained by performing tissue acquisition necropsy at the University of Washington [20,128,129,130,131,132,133,134,135,136,137,138,139,140]. During 1991–2005, 261 PCa samples were collected from 156 patients and were implanted subcutaneously into male SCID mice. Of these, 26 samples were successfully propagated beyond three passages, with an overall integration rate of ∼10%. The LuCaP PDX models show the major genomic and phenotypic features of PCa specimens obtained from patients, including AR amplification, PTEN deletion, TP53 deletion and mutation, RB1 loss, TMPRSS-ERG rearrangement, SPOP mutation, hypermutation due to MSH2/MSH6 genomic aberrations, and BRCA2 loss. Moreover, the heterogeneity of the response of the LuCaP PDX models to androgen deprivation and docetaxel is similar to that of patients to these treatments. PDX models LuCaP 23.1 and LuCaP 35 were derived from patient-derived cell lines LuCap 23.1 and LuCaP 35, respectively [128,129,130]. LuCaP 49 PDX model was derived from an NEPC specimen obtained from a 71-year-old man with clinical stage B-II prostate carcinoma [135]. The LuCaP PDX models are abiraterone resistant [136]. CRPC PDX models LuCaP 136CR, 77CR, 96CR, and 35CR PDXs treated with abiraterone have been used to evaluate mechanisms underlying response and resistance to abiraterone. Although abiraterone treatment significantly inhibits the proliferation of LuCaP 136CR-derived tumors, it does not inhibit or only minimally inhibits the proliferation of LuCaP 35CR-derived tumors. The molecular signature of secreted proteins associated with an abiraterone ultra-responsive phenotype has been determined [132]. The CRPC PDX models can be used to evaluate new drugs targeting histone acetyltransferase paralogue p300 and CREB-binding protein [137,138]. Whole-genome microarray analysis of the LuCaP PDX models has indicated that a neuroendocrine CRPC molecular phenotype is defined by the dual positivity of CgA and synaptophysin, and is not necessarily associated with the loss of AR activity [134]. Two clinically relevant AR splice variants, namely, ARv567 and ARv7, are differentially expressed in the LuCaP PDX models [139,140]. This difference can be used to determine taxane sensitivity of PCa [140].

The LAPC PDX models were first reported in 1997 by the UCLA School of Medicine [63] and were developed by subcutaneously transplanting metastatic PCa specimens into SCID mice. The LAPC-4 cell line has been derived from a LAPC-4 xenograft model (see cell line section). LAPC-9 PDX model expresses PSA and WT AR, undergoes growth arrest in response to castration, and exists in a dormant, androgen-responsive state for at least 6 months. After prolonged androgen deprivation, the LAPC-9 PDX model shows a spontaneous androgen-independent outgrowth [141].

The BM18 PDX model was derived in 2005 by subcutaneously xenografting a metastatic PCa tissue, which was curetted from the femoral bone of an 88-year-old man who underwent intramedullary fixation for a pending fracture, into SCID mice [142]. The BM18 PDX model shows positivity for PSA, AR, CK-18, and pan keratin consistent with that observed in the original patient tumor specimen. Androgen-deprivation induces the regression of tumors derived from this PDX model. Moreover, the sizes of tumors derived from the BM18 PDX model are positively correlated with serum PSA levels in mice.

The Living Tumor Laboratory (LTL) PDX models were established from adenocarcinoma or NEPC tissues obtained from patients at Vancouver Prostate Centre [143,144,145,146,147,148,149,150]. They release the information of LTL PDX lines at their web site (www.livingtumorlab.com). They report that although survival rate (1st generation xenografts) in PCa is > 95%, the success rate in establishing PCa tissue lines (≥ 5 generations) is 17% (10 of 59). LTL-311 PDX model was first established in 2008 by grafting the tissues under the kidney capsules of NOD/SCID mice. The LTL PDX models include varying types of PCa, hormone-naïve adenocarcinoma, CRPC, and NEPC cells. LTL-352 PDX model was derived from an NEPC patient specimen and expresses an NE marker but not PSA. LTL-331R-G7 PDX model was derived from an adenocarcinoma specimen and shows neuroendocrine features after host castration and passaging. These NEPC models show significant suppression of FOLH1, amplification of SSTR2, and abnormal expression of heterochromatin genes [143,150]. Gene expression microarray analysis of LTL-313H and LTL-313B PDX models, which were derived from a single PCa patient and show different metastatic potentials, resulted in the identification of the candidate biomarker gene TMEM45B. Increased TMEM45B expression is suggested to be significantly associated with PCa progression and metastasis, thus providing a new prognostic biomarker for predicting the metastasis [144]. The LTL PDX models can be used to identify metabolic heterogeneity signatures [146], CRPC driver genes [145], stroma-derived metastasis signatures [148], and new drug efficacies [149].

The KUCaP-2 PDX model was derived in 2010 from trans-urethrally resected specimens of local recurrent tumors obtained from a Japanese patient by performing radical prostatectomy [151]. The specimens were minced into 20- to 30-mm^3^ pieces and subcutaneously transplanted into male nude mice along with 50 μL Matrigel^TM^. The KUCaP-2 xenograft was established ∼10 months after the first inoculation. These xenografts express WT AR and PSA. Tumors derived from the KUCaP-2 PDX model regress immediately after castration but recur after 1–2 months, thus mimicking the clinical behavior of CRPC.

The MDA PCa PDX models were derived from primary or metastatic PCa specimens obtained from operative specimens available at The University of Texas MD Anderson Cancer Center [152,153,154,155,156,157]. Information on these PDX models is summarized in a study by Li et al. [152]. MDA PCa 144 model was derived from an NEPC specimen that was obtained by performing salvage pelvic exenteration from a 72-year-old man who was treated with androgen deprivation therapy, external-beam radiation, and carboplatin and docetaxel combination therapy [153,154]. MDA PCa 155 model shows the features of NEPC [154]. DNA methylation profiles of AR-negative and AR-positive MDA PCa PDX models were compared using methylated CpG island amplification and microarray analysis to identify differentially methylated promoters in these models and the corresponding donor patient samples. The AR promoter in the MDA PCa PDX models shows silencing histone modifications (H3K27me3 and H3K9me2); moreover, EZH2 inhibition with 3-deazaneplanocin A induces AR expression and growth inhibition in the AR-negative MDA PCa PDX models [155]. MDA PCa 118b model was generated from an osteoblastic bone lesion and produces osteoblastic tumors upon subcutaneous implantation or implantation into mouse femurs [156]. The MDA PCa 118b model can be used to determine a complex PCa bone metastasis secretome with paracrine and autocrine signaling functions that mediates a cross-talk among multiple cell types within the tumor microenvironment. Poorly adherent MDA PCa PDX tumor cells exhibit low viability in standard culture, thus making it difficult to manipulate these cells for subsequent controlled mechanistic studies. Establishment of three-dimensional cultures by using hyaluronan-based hydrogels helps in maintaining cell viability in the MDA PCa PDX models, with continued native AR expression. This culture method is useful for rapid drug evaluation, thus making personalized medicine a clinical reality [157].

The Monash University PDX models were derived using localized primary PCa and CRPC specimens available at the Monash University. The xenograft success rate of localized primary PCa tissues was improved using chimeric grafts with neonatal mouse mesenchyme. The use of the neonatal mesenchyme significantly increased the xenograft survival rate and doubled the proliferation index of xenografted cancer cells [124]. The mouse mesenchyme can be replaced with human prostate fibroblasts to determine their involvement in tumor progression [158]. CRPC PDX models were established by grafting CRPC tissues under the renal capsule of NOD/SCID or NSG mice [159,160]. For performing castration experiments, the PDXs were re-grafted into testosterone-implanted mice, followed by the castration of 50% mice and removal of their testosterone implants. Ten new serially transplantable PDXs were established by collecting 109 tumor samples from 29 patients with CRPC. Of these CRPC PDXs, PDX-27 and PDX-201 were derived from abiraterone- and ENZ-resistant tumors, respectively [160]. Integrative genomic analysis indicated that these CRPC PDXs showed heterogeneous mechanisms of resistance, including known and novel AR mutations, genomic structural rearrangements in the AR gene, and neuroendocrine-like AR-null phenotype. These PDXs were used to evaluate the relevance of intraductal carcinoma of the prostate (IDC-P) in advanced PCa [161]. The volume of IDC-P derived from these PDXs was similar to that of adenocarcinoma. Castration resulted in the persistence of IDC-P lesions, and testosterone restoration led to tumor regeneration similar to that observed for castration-resistant adenocarcinoma.

## 4. Three-Dimensional Cultures of Patient-Derived PCa Cells

Although the use of PDX models helps in overcoming the limitations associated with the use of cell lines, the PDX models are associated with some limitations. Engraftment and drug validation in mice usually requires >6 months. This time delay limits the applicability of PDX models in real-time patient treatment. Moreover, although PDX models are suitable for examining a limited number of drug combinations, they are not amenable to high-throughput screening [17]. Therefore, three-dimensional cultures are attracting considerable attention as patient-derived cancer models. Three-dimensional organoid culture models closely recapitulate the heterogeneous genetic and morphological features of original tumors. Organoid models of patient-derived cancer cells were initially established for colorectal cancer (CRC). The idea to culture primary CRC cells for a long period came from the fundamental discovery that healthy intestinal stem cells can be cultured using Wnt, R-spondin1, EGF, and noggin [27,171,172]. Healthy intestinal stem cells form organoids in Matrigel^TM^ and retain their normal genome over time [173]. Matrigel^TM^ and a cocktail of essential stem cell growth factors supplemented with a transforming growth factor-β receptor inhibitor (A83-01) and p38 MAPK inhibitor (SB202190) can be used as a growth medium for culturing healthy human intestinal or colon cells, and eventually for culturing CRC organoids [27]. Thereafter, similar culture protocols have been developed for pancreatic cancer [174] and PCa [29]. Organoid models of PCa retain parental tumor heterogeneity and show improved efficacy. Another three-dimensional culture model, i.e., spheroid culture model, is associated with a high success rate among other patient-derived cell culture models. Spheroid culture models are established using ultra-low-attachment culture plates. Moreover, Rho-associated protein kinase inhibitor Y-27632 greatly facilitates the establishment of spheroid culture models [175,176]. Linxweiler et al. reported spheroid models generated from radical prostatectomy specimens of organ-confined PCa [177]. Although the idea of a three-dimensional PCa culture is in its nascent stage, it has the potential to improve drug development and clinical application. In this section, we have summarized a few PCa organoid and spheroid models derived from patients with PCa.

The MSK-PCa model was the first reported patient-derived organoid model of PCa, developed in 2014 by Memorial Sloan Kettering Cancer Center [29], and was established from biopsy specimens of PCa metastases and circulating tumor cells. Fresh tissue biopsy samples of metastatic PCa were mechanically dissected and enzymatically digested. Circulating tumor cells were prepared by incubating patient blood samples with RosetteSep^®^ Human CD45 Depletion Cocktail, followed by the depletion of red and white blood cells by using Ficoll-Paque. Next, the cells were washed, placed in ice-cold Matrigel^TM^, and plated in the middle of one well of a 24-well plate containing human prostate culture medium. The prostate-specific culture medium contained DMEM/F12 supplemented with GlutaMAX, penicillin, streptomycin, primocin, B27, *N*-acetylcysteine, mouse recombinant EGF, human recombinant FGF-10, FGF-basic, A-83-01, SB202190, nicotinamide, testosterone, PGE2, Y-27632, noggin-conditioned medium, and R-spondin-conditioned medium [29,178]. The efficiency of establishing a continuously propagated organoid model from metastatic biopsies is 15–20% (6/32). These models form tumors when grafted into SCID mice. The MSK-PCa organoid model recapitulates the molecular diversity of different PCa subtypes. Whole-exome sequencing has shown that the MSK-PCa organoid model has a low mutational burden.

The ORG WCM organoid model was established in 2018 from a biopsy specimen of metastatic PCa obtained from patients with NEPC at Weill Cornell Medicine [179]. For this, fresh tissue biopsy samples of metastatic PCa were mechanically dissected and enzymatically digested. Next, the cells obtained were resuspended in a prostate-specific culture medium [178]. The resuspended pellet was combined with growth factor-reduced Matrigel^TM^ in a 1:2 volume ratio. Fresh tumor tissues from 25 patients with metastatic PCa were used for developing organoid models, with an overall success rate of 16% (4/25). The organoids models were engrafted as patient-derived organoid xenografts (PDOXs) into NOD/SCID mice and were subsequently re-passaged in vitro as organoids from PDOXs. The metastatic tumors obtained from each of the four patients and their matched organoids and PDOXs were classified as NEPC based on their tumor morphology. The organoids and PDOXs were clustered based on the shared expression of CRPC-NE signature genes, including the overexpression of MYCN, PEG10, SRRM4, EZH2, SOX2, BRN2, and FOXA2 genes, and decreased expression of AR signaling genes. These organoid models were used to confirm the effect of EZH2 inhibitor on NEPC.

The Saarland University spheroid models were generated in 2018 from radical prostatectomy specimens of organ-confined PCa [177]. For this, the tissue samples were mechanically dissected, enzymatically digested, and filtered. Next, the cells were resuspended in a modified stem cell medium (StemPro medium) and cultured in ultra-low-attachment multiwell plates. The StemPro medium contained GlutaMAX, DMEM/F-12, BSA, 2-mercaptoethanol, StemPro^®^ hESC supplement, penicillin/streptomycin, R1881, FGF-basic, and Y-27632 dihydrochloride. In all, 109 spheroid models were established using tissues obtained from 173 patients. These models stayed viable for several months and were amenable to cryopreservation. Almost all the models showed AR, CK8, and AMACR positivity. The viability of these spheroid models markedly reduced upon bicalutamide and ENZ treatment. However, abiraterone treatment had no effect and docetaxel treatment only had a moderate effect on these models.

## 5. Conclusions

In the last several decades, quantitative analysis of molecular studies has been advanced in the field of prostate cancer research based on the establishment of PCa cell lines and their drug-resistant sublines. A small number of cell lines, however, could be established from cancer tissues with rare possibilities, and their application to preclinical studies was rather limited due to their characteristics usually being apart from the original tumors. Recent advances in culture technology and mouse engineering has improved the success rates for the establishment of patient-derived cancer platforms, including PDC organoid/spheroid and PDX models, which retain the characteristics of the original tumors and tumor heterogeneity. The generation of PDC models is currently still challenging, particularly from primary hormone-naïve PCa, nevertheless, it has preclinical relevance, as PDCs have advantages for large-scale drug screening and its application for PDX reestablishment. Considering current clinical streams towards cancer precision medicine, patient-derived cancer models are powerful and promising “avatars” of the original tumors, and these PCa platforms are favorable for the characterization of the clinical pathophysiology and the determination of the most efficient therapeutic regimen.

## Figures and Tables

**Table 1:** Established prostate cancer cell lines.

Name	Pathology	Origin	Race	Pretreatment	AR	PSA	First Report Year	References
1013L	Adeno	primary	unknown	none	-	-	1980	[33,34,35,36,37]
E006AA	Adeno	primary	AA	none	+	±	2004	[38,39,40]
RC-77T/E	Adeno	primary	AA	none	+	+	2010	[41,42]
DU-145	Adeno	metastasis	Caucasian	none	-	-	1975	[10,43,44,45,46,47]
PC-3	Adeno	metastasis	Caucasian	none	-	-	1979	[11,48,49,50,51,52,53]
LNCaP	Adeno	metastasis	Caucasian	none	+	+	1980	[9,12,54,55,56,57]
ARCaP	Adeno	metastasis	Caucasian	none	±	±	1996	[58]
MDA PCA 2a/b	Adeno	metastasis	AA	ADT	±	+	1997	[59,60]
LuCap 23	Adeno	xenograft tumor from metastasis	Caucasian	ADT, chemotherapy	+	+	1996	[46,61,62]
LAPC-4	Adeno	xenograft tumor from metastasis	Caucasian	ADT	+	+	1997	[63,64,65]
22Rv1	Adeno	xenograft tumor from primary tumor	Caucasian	none	+	+	1999	[2,66,67,68]
VCaP	Adeno	xenograft tumor from metastasis	Caucasian	unknown	+	+	2001	[69,70]
KUCaP	Adeno	xenograft tumor from metastasis	Asian	ADT	+	+	2005	[71,72,73,74]
PC346	Adeno	xenograft tumor from primary tumor	Caucasian	ADT	+	+	2006	[75]

Adeno: adenocarcinoma, AA: African American, ADT: androgen deprivation therapy.

**Table 2:** Treatment-resistant sublines derived from prostate cancer cell lines.

Name	Character	Parent Cells	Treatment	Method	First Report Year	References
LNCaP-abl	Cas R	LNCaP	castration	culture in androgen depleted medium	1999	[32]
LNCaP-SF	Cas R	LNCaP	castration	culture in androgen depleted medium	2003	[76,77]
LNCaP-LTAD	Cas R	LNCaP	castration	culture in androgen depleted medium	2012	[14,78,79]
C4-2	Cas R	LNCaP	castration	derived from xenograft tumor in castrated mouse	1994	[80,81]
PC346Flu1/2	AA R	PC346	castration and flutamide	culture in androgen depleted medium with flutamide	2011	[82]
LNCaP-BicR (Takayama)	AA R	LNCaP	bicalutamide	culture with flutamide	2015	[13]
LNCaP-BicR (Liu)	AA R	LNCaP	bicalutamide	culture with flutamide	2017	[83]
MR49F	AA R	LNCaP	enzalutamide	derived from xenograft tumor treated with enzalutamide	2013	[84,85]
ENZ^R^ cell line series	AA R, NEPC	LNCaP	enzalutamide	derived from xenograft tumor treated with enzalutamide	2017	[86]
DU145-TxR	Chemo R	DU145	paclitaxel	culture with paclitaxel	2007	[87,88,89,90,91,92,93,94,95,96]
PC-3-TxR	Chemo R	PC-3	paclitaxel	culture with paclitaxel	2007	[87,88,89,90,91,92,93,94,95,96]
PC-3PR	Chemo R	PC-3	paclitaxel	culture with paclitaxel	2010	[97,98,99,100]
PC-3-Pa	Chemo R	PC-3	paclitaxel	culture with paclitaxel	2018	[101]
PC-3dR	Chemo R	PC-3	docetaxel	culture with docetaxel	2008	[102]
DU145R (O’Neill)	Chemo R	DU145	docetaxel	culture with docetaxel	2011	[103,104]
22Rv1R	Chemo R	22Rv1	docetaxel	culture with docetaxel	2011	[103,104]
PC-3 D12	Chemo R	PC-3	docetaxel	culture with docetaxel	2011	[103,104]
DU145R (Marin)	Chemo R	DU145	docetaxel	culture with docetaxel	2012	[105]
PC-3R	Chemo R	PC-3	docetaxel	culture with docetaxel	2012	[105]
DU145CR	Chemo R	DU145	cabazitaxel	culture with cabazitaxel	2018	[106]
PC-3CR	Chemo R	PC-3	cabazitaxel	culture with cabazitaxel	2018	[106]
DU145-TxR/CxR	Chemo R	DU145-TxR	cabazitaxel	culture with cabazitaxel	2018	[107]
PC-3-TxR/CxR	Chemo R	PC-3-TxR	cabazitaxel	culture with cabazitaxel	2018	[107]

Cas R: castration resistant, AA R: anti-androgen agent resistant, Chemo R: chemotherapy resistant, NEPC: neuroendocrine prostate cancer.

**Table 3:** Patient-derived xenografts of prostate cancer.

Name	Pathology	Origin	Host Mouse	Method	First Report Year	References
Rotterdam PC-models	Adeno, NEPC	primary, metastasis	Athymic Nude	SC	1977	[22,75,121,125,126,127]
LuCaP series	Adeno, NEPC	primary, metastasis	SCID	SC	1991	[20,128,129,130,131,132,133,134,135,136,137,138,139,140]
LAPC-series	Adeno	metastasis	SCID	SC	1997	[63,141]
BM18	Adeno	metastasis	SCID	SC	2005	[142]
LTL-series	Adeno, NEPC	primary, metastasis	NOD/SCID	SR	2008	[143,144,145,146,147,148,149,150]
KuCaP-2	Adeno	local recurrent	Athymic Nude	SC	2010	[151]
MDA Pca series	Adeno, NEPC	primary, metastasis	SCID	SC	2011	[152,153,154,155,156,157]
Monash University PDX series	Adeno, NEPC	primary, metastasis	NOD/SCID, NSG	SR	2011	[124,158,159,160,161]

Adeno: adenocarcinoma, NEPC: neuroendocrine prostate cancer, SC: subcutaneous, SR: subrenal.
